# Case for diagnosis. Bilateral ulcerations on the distal phalanges of the second and third fingers - Ulcerative carpal tunnel syndrome^☆^

**DOI:** 10.1016/j.abd.2022.03.008

**Published:** 2023-03-10

**Authors:** Hiram Larangeira de Almeida Jr., Fernanda Pinto Garcia, Laura de Moraes Gomes, Antônia Larangeira de Almeida

**Affiliations:** a Department of Dermatology, Universidade Católica de Pelotas, Pelotas, RS, Brazil; b Department of Dermatology, Universidade Federal de Pelotas, Pelotas, RS, Brazil; c Hospital Erasto Gaertner, Curitiba, RS, Brazil; d Department of Radiology, Universidade Federal de Pelotas, Pelotas, RS, Brazil

Dear Editor,

A 60-year-old female, hypertensive, type 2 diabetic patient with dyslipidemia, who worked in gastronomy, reported bilateral paresthesia in the thumb, index and middle fingers associated with nocturnal pain, which had started three years before. Two years before, she started to present ulcerated, bleeding, painless skin lesions on the second and third fingers, whereas on the third finger of the left hand, she had a healed lesion (Fig. 1A and B). The patient did not have any other skin lesions or other signs suggestive of leprosy, such as neural thickening, or loss of strength in other muscle groups. She also had hypoesthesia in these fingers, reporting no pain from professional injuries.

**Figure 1:**
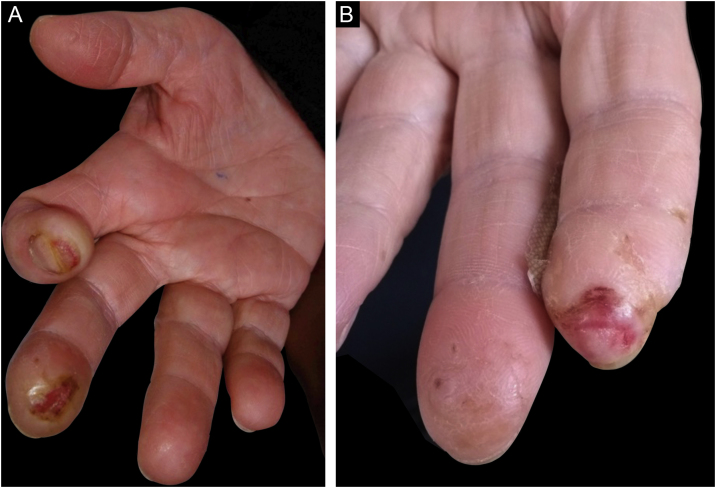
Clinical aspect. (A) Right hand - subungual ulceration on the index finger, ulcer with a callus border on the distal phalanx of the middle finger, and atrophy of the thenar region. (B) Left hand - ulceration on the distal phalanx of the index finger and decrease in the size of the phalanx; there is also a healed area on the distal phalanx of the middle finger.

Neurological examination showed that Tinel’s sign was present, as well as pain on wrist flexion. Electroneuromyography showed severe sensorimotor axonal neuropathy of the median nerve, which affected its distal segment, with an absence of potential in the nerve conduction test. X-rays of the hands showed osteolysis of the distal phalanx of the index fingers. A wrist magnetic resonance imaging showed bilateral thickening of the median nerve in the retinaculum (Fig. 2A and B), with normal thickness outside this region (Fig. 2C), whereas the ulnar nerves showed a normal appearance.

**Figure 2:**
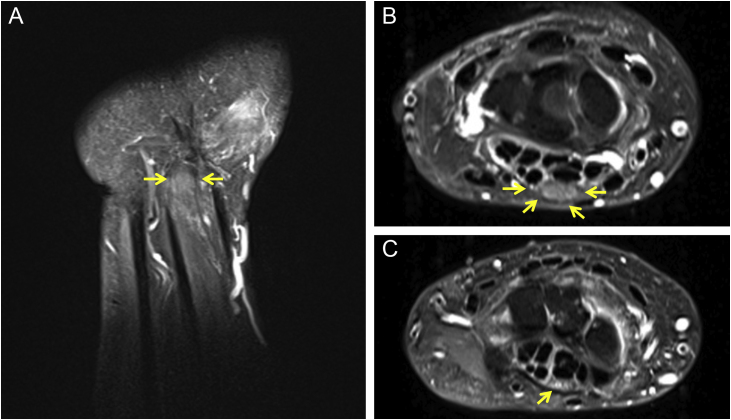
Magnetic resonance imaging of the right wrist. (A) Sagittal view showing thickening of the median nerve (arrows). (B) Axial view showing thickening of the median nerve in the carpal tunnel (arrows). (C) Nerve showing normal thickness outside the carpal tunnel (arrow).

## What’s your diagnosis?

a) Leprosy
b) Diabetic neuropathy
c) Ulcerative carpal tunnel syndrome
d) Dermatitis artefacta

## Discussion

Carpal tunnel syndrome (CTS) is a focal mononeuropathy caused by compression of the median nerve as it goes through the osteofibrous carpal tunnel. In the case reported herein, the nerve measured 18.7 mm^2^ in the left retinaculum and 22.1 mm^2^ in the right one, with normal size being up to 15 mm^2^; measurements above 19 mm^2^ are considered a severe form of the disease.^1^

The idiopathic form of CTS occurs more often in women aged between 40 and 60 years, and in half of the cases it is bilateral,^2^ similar to the case described herein.

Secondary forms are caused by trauma, such as dislocation of the carpal bones, or by joint alterations in the wrist, such as osteoarthritis or inflammatory arthritis.^2^

Cutaneous involvement in CTS is rare, having been described as an ulcerative-mutilating form in 1979 by Bouvier,^3^ with some reports in the dermatological literature,^4^, ^5^, ^6^ which are generally described as ulcerative or ulcerative-mutilating forms.

There is a Portuguese report of nine cases in a retrospective study,^7^ confirmed by electromyography. All of them started with paresthesia in the second and third fingers and one case had nocturnal pain. All radiographed cases (one-third) had osteolysis of the distal phalanges. Seven cases (77%) had ulcerations on the second and third fingers, also similar to the case described herein, and two cases had ulceration in only one finger.

The lesions observed in this patient are characteristic of peripheral neuropathy, with a clear background and callused edges and without pain, probably aggravated by the patient’s professional activity. CTS should be suspected whenever there is involvement restricted to the second and third fingers, and the thumb is usually spared, as it has combined innervation of the radial nerve.^7^

It is relevant in dermatology to know about this disease, given the important differential diagnosis with leprosy (although it cannot be completely ruled out), so these cases can be referred for orthopedic evaluation and treatment, which can be conservative or surgical, with retinaculotomy, which gave good results in this patient..^8^

## Financial support

None declared.

## Authors’ contributions

Hiram Larangeira de Almeida Jr.: Approval of the final version of the manuscript; design and planning of the study; drafting and editing of the manuscript; collection, analysis and interpretation of data; intellectual participation in the propaedeutic and/or therapeutic conduct of the studied cases; critical review of the literature; critical review of the manuscript.

Fernanda Pinto Garcia: Approval of the final version of the manuscript; design and planning of the study; drafting and editing of the manuscript; collection, analysis and interpretation of data; intellectual participation in the propaedeutic and/or therapeutic conduct of the studied cases; critical review of the literature; critical review of the manuscript.

Laura de Moraes Gomes: Approval of the final version of the manuscript; drafting and editing of the manuscript; collection, analysis and interpretation of data; intellectual participation in the propaedeutic and/or therapeutic conduct of the studied cases; critical review of the literature; critical review of the manuscript.

Antônia Larangeira de Almeida: Approval of the final version of the manuscript; design and planning of the study; drafting and editing of the manuscript; collection, analysis and interpretation of data; critical review of the literature; critical review of the manuscript.

## Conflicts of interest

None declared.
